# Synovial interleukin‐6 point‐of‐care testing, alone and combined with D‐dimer, supports rapid diagnosis of periprosthetic joint infection

**DOI:** 10.1002/ksa.70289

**Published:** 2026-01-19

**Authors:** Sujeesh Sebastian, Hibah Abdulwahab Abusulaiman, Veronika Achatz, Matteo Spadini, Tobias Josef Niedrist, Markus Herrmann, Jochen Gerhard Hofstaetter

**Affiliations:** ^1^ Michael Ogon Laboratory for Orthopaedic Research, Orthopaedic Hospital Vienna‐Speising Vienna Austria; ^2^ Department of Orthopaedics and Trauma Medical University of Graz Graz Austria; ^3^ Clinical Institute of Medical and Chemical Laboratory Diagnostics Medical University of Graz Graz Austria; ^4^ 2nd Department Orthopaedic Hospital Vienna‐Speising Vienna Austria; ^5^ Medical University of Vienna Vienna Austria

**Keywords:** arthroplasty, D‐dimer, interleukin‐6, procalcitonin, prosthetic joint infection, synovial fluid

## Abstract

**Purpose:**

Data on the reliability of point‐of‐care (POC) tests for the rapid diagnosis of periprosthetic joint infections (PJI) using synovial fluid (SF) D‐dimer (DD), interleukin 6 (IL‐6) and procalcitonin (PCT) are limited. This study aimed to address this issue by evaluating the diagnostic accuracy of POC tests for SF DD, IL‐6 and PCT in comparison to standard laboratory devices for diagnosing PJI.

**Methods:**

A total of 273 patients who underwent 300 revision arthroplasties were included in this study. According to the European Bone and Joint Infection criteria, 123 were classified as infected, 145 as aseptic revisions and 32 as likely infected. SF DD, IL‐6 and PCT levels were quantified using both POC and standard laboratory tests. The diagnostic accuracy was assessed using receiver operating characteristic curves and area under the curve (AUC).

**Results:**

Among POC tests, IL‐6 demonstrated the highest AUC 0.83 (95% confidence interval CI 0.77–0.89), followed by DD (0.66, 95% CI 0.59–0.72), while PCT performed poorly (AUC 0.26, 95% CI 0.20–0.33). Laboratory‐based IL‐6 test outperformed all other markers (AUC 0.94, 95% CI 0.90–0.97). When using both POC and standard laboratory methods, significant differences were noted for IL‐6 and DD (*p* < 0.001). Combining the POC test results for IL‐6 and DD showed better diagnostic performance than either alone (AUC 0.92). In patients with PJI, biomarker levels differed by pathogen virulence, except for POC IL‐6 (*p* = 0.631), POC DD (*p* = 0.080) and laboratory DD (*p* = 0.053).

**Conclusions:**

POC tests for SF, particularly those for IL‐6, support the rapid PJI diagnosis, and combining POC IL‑6 with DD further improves diagnostic accuracy. These findings suggest that POC IL‐6, either independently or in conjunction with DD, could be useful in situations where rapid decisions are required. However, its inferior performance compared to that of laboratory assays highlights the need for further optimisation and validation.

**Level of Evidence:**

Level II, development of diagnostic criteria in a consecutive series of patients and a universally applied ‘gold’ standard.

**Abbreviations**
AD: alpha‐defensin
AUC: area under the curve
CRP: C‐reactive protein
DD: D‐dimer
EBJIS: European Bone and Joint infection society
IL‐6: interleukin‐6
LE: leucocyte esterase
PCT: procalcitonin
PJI: periprosthetic joint infections
PMN%: polymorphonuclear leucocyte percentage
POC: point‐of‐care
ROC: receiver operating characteristic
rTHA: revision total hip arthroplasty
rTKA: revision total knee arthroplasty
SF: synovial fluid
UNIC: unexpected negative intraoperative culture
WBC: white blood cell

## INTRODUCTION

Synovial fluid (SF) leucocyte count, polymorphonuclear leucocyte percentage and culture analysis are standard tests for diagnosing periprosthetic joint infection (PJI) [19]. However, conventional laboratory analyses are often time‐consuming and have limited applicability in outpatient and ambulatory settings. Point‑of‑care (POC) tests, which provide rapid results, represent a promising strategy for improving PJI diagnosis [12]. Although alpha‐defensin and leucocyte esterase are among the few SF POC tests currently recommended [19, 24], their clinical use is limited by high costs and interference from blood contamination in the samples [28, 30]. While synovial interleukin‐6 (IL‐6) has demonstrated diagnostic utility primarily through conventional laboratory assays [16, 31, 32], D‐dimer (DD) and procalcitonin (PCT) remain unexplored as POC tests for PJI [4, 13]. These markers were selected for POC testing based on their complementary roles and diverse pathophysiological pathways [4, 13, 16, 31, 32].

Patients presenting with unexpected negative intraoperative cultures (UNICs), ambiguous biomarker levels relative to pathogen virulence, implant‐associated complications such as metallosis or polyethylene abrasion, or suspected PJI cases, where recommended markers are within normal ranges, pose diagnostic challenges in revision arthroplasties [18, 22]. However, the diagnostic efficacy of POC tests in these settings is poorly characterised [4, 13, 16, 18, 31, 32].

The present study aimed to (a) evaluate the diagnostic accuracy of SF POC tests for DD, IL‑6 and PCT compared with standard laboratory assays and (b) assess their performance in diagnostically challenging scenarios, including UNICs, infections with low‑virulent pathogens, metallosis or polyethylene wear and confirmed PJIs with normal routine markers. We hypothesised that synovial POC tests, particularly for IL‐6, would achieve diagnostic accuracy comparable to that of laboratory assays and that the combination of POC IL‐6 with DD would further enhance performance in complex clinical situations. Unlike previous studies that have evaluated these markers predominantly using laboratory assays, this study systematically compared POC IL‑6, DD and PCT in revision arthroplasties [4, 13, 16]. By focusing on rapid and relatively affordable POC assays, this study addresses the need for more effective outpatient and intraoperative PJI diagnoses.

## MATERIALS AND METHODS

### Study design and patient selection

A single‐centre retrospective diagnostic accuracy study was conducted using our prospectively collected institutional arthroplasty registry and musculoskeletal biobank from October 2019 to October 2024. The study population comprised all patients who underwent preoperative SF aspiration as part of the diagnostic workup for PJI prior to revision total hip and knee arthroplasty (THA and TKA). The European Bone and Joint Infection Society (EBJIS) criteria were used to classify the procedures as ‘infection confirmed’, ‘likely’ or ‘unlikely’ [19]. Clinical and baseline demographic data were collected for all patients, including age, sex, body mass index, arthroplasty localisation, preoperative laboratory values (serum C‐reactive protein, SF white blood cell count and polymorphonuclear percentage), number of prior revision surgeries, surgical details, microbiology and pathology reports. Patients who underwent reimplantation or spacer exchange were excluded.

### Study definitions

Infection‐confirmed cases based on EBJIS criteria, in which all intraoperative culture results were negative but one or more positive findings, such as the presence of clinical features like sinus tract, raised serum/synovial markers, purulence around the prosthesis or a positive histological finding, were classified as UNICs. High‐virulent pathogens included *Staphylococcus aureus*, *Staphylococcus lugdunensis*, *Streptococci*, *Enterococci*, *Enterobacteriaceae* and nonfermenting Gram‐negative bacilli. Pathogens not classified as highly virulent were designated as low‐virulent pathogens [23]. The cause of revision surgery was attributed to metallosis or polyethylene abrasion if documented by the operating surgeon in the operative notes.

### Study population and revision arthroplasty characteristics

A total of 273 patients who underwent 300 revision arthroplasties were included in the final analysis (Table 1). Of these 300 revision arthroplasties, 123 were classified as infected, 145 as aseptic revisions and 32 as likely infected according to the EBJIS criteria. Among the septic revisions, 23 (18.7%) had UNIC results. In the 100 culture‐positive septic revisions, a high‐virulent pathogen was identified in 60/100 (60%) procedures, whereas in 40/100 (40%) procedures, low‐virulent pathogens were identified. The most frequently isolated pathogen in septic revisions was *Staphylococcus epidermidis* (20%) (Table 2).

**Table 1:** Demographic and clinical data.

	Total	rTHA	rTKA
Total procedures, *n* (%)	300	138 (46.0)	162 (54.0)
Sex, *n* (%)			
Female	169 (61.9)	78 (60.0)	91 (63.6)
Male	104 (38.1)	52 (40.0)	52 (36.4)
Total patients	273	130	143
Age in years (mean ± SD)	68.7 ± 12.9	68.3 ± 14.9	69 ± 10.8
BMI (mean ± SD)	29.7 ± 6.7	28.4 ± 5.7	30.8 ± 7.3
Serum and synovial inflammatory markers (median and IQR)			
Serum CRP (mg/L)	6.8 (2.4–44.7)	6.1 (2.1–30.3)	10 (2.5–69.3)
Synovial WBC count (cells/µL)	1530 (430–27,490)	1740 (610–22,260)	1325 (325–35,555)
Synovial PMN (%)	47.8 (21.8–87.6)	50.7 (27.7–83.7)	43.1 (21.4–88.2)
EBJIS PJI classification			
Infection confirmed	123 (41.0)	53 (38.4)	70 (43.2)
Infection likely	32 (10.7)	18 (13.0)	14 (8.6)
Infection unlikely	145 (48.3)	67 (48.6)	78 (48.1)
On antibiotic therapy at the time of aspiration, *n* (%)	43 (14.4)	18 (13.0)	25(15.5)
Type of revision			
1st revision	148 (49.3)	70 (50.7)	78 (48.1)
Re‐revision	152 (50.7)	68 (49.3)	84 (51.9)
Time to previous surgery			
<90 days (acute procedure)	47 (15.7)	31 (22.5)	16 (9.9)
>90 days (chronic procedure)	253 (84.3)	107 (77.5)	146 (90.1)
Reason for revision surgery			
Infection	104 (34.7)	42 (30.4)	62 (38.3)
Loosening	76 (25.3)	51 (37.0)	25 (15.4)
Instability/dislocation	48 (16.0)	23 (16.7)	25 (15.4)
Metallosis	16 (5.3)	8 (5.8)	8 (4.9)
Pain	15 (5.0)	3 (2.2)	12 (7.4)
Polyethylene abrasion	12 (4.0)	2 (1.4)	10 (6.2)
Periprosthetic fracture	11 (3.7)	9 (6.5)	2 (1.2)
Quadriceps tendon rupture	10 (3.3)	0	10 (6.2)
Patella resurfacing	7 (2.3)	0	7 (4.3)
Arthrofibrosis	1 (0.3)	0	1 (0.6)
ASA score, *n* (%)			
ASA I	24 (8.8)	17 (13.1)	7 (4.9)
ASA II	186 (68.1)	87 (66.9)	99 (69.2)
ASA III	63 (23.1)	26 (20.0)	37 (25.9)
Charlson comorbidity index, median (range)	3 (0–10)	3 (0–10)	4 (0–10)

Abbreviations: ASA, American Society of Anesthesiologists; BMI, body mass index; CRP, C‐reactive protein; IQR, interquartile range; PMN, polymorphonuclear leucocytes; rTHA, revision total hip arthroplasty; rTKA, revision total knee arthroplasty; SD, standard deviation; WBC, white blood cell.

**Table 2:** Microorganisms detected in infected total hip and knee arthroplasties.

Procedures diagnosed as infected	*N* = 123
Culture negative	23 (18.7%)
Culture positive	100 (81.3%)
Monomicobial infection	79 (79%)
Polymicrobial infection	21 (21%)
Microorganisms, total	127
Aerobic gram‐positive, *n* (%)	78 (61.4%)
*Staphylococcus epidermidis*	25
*Staphylococcus aureus*	15
*Staphylococcus lugdunensis*	7
*Staphylococcus capitis*	4
*Staphylococcus hominis*	4
*Staphylococcus caprae*	1
*Staphylococcus saprophyticus*	1
*Staphylococcus petrasii*	1
*Streptococcus agalactiae*	11
*Streptococcus dysgalactiae*	4
*Other Streptococcus spp.*	1
*Enterococcus faecalis*	3
*Granulicatella adiacens*	1
Aerobic gram‐negative, *n* (%)	26 (20.6%)
*Escherichia coli*	8
*Proteus mirabilis*	7
*Citrobacter koseri*	3
*Pseudomonas aeruginosa*	1
*Enterobacter cloacae*	1
*Enterobacter ludwigii*	1
*Morgenella morganii*	1
*Salmonella spp.*	1
*Raoultella ornithinolytica*	1
*Serratia marcescens*	1
*Roseomonas mucosa*	1
Anaerobes, *n* (%)	12 (9.4%)
*Cutibacterium avidum*	7
*Cutibacterium acnes*	5
Other gram‐positive bacteria	6 (4.7%)
Fungi, *n* (%)	5 (3.9%)
*Candida glabrata*	2
*Candida parapsilosis*	1
*Candida albicans*	1
*Candida krusei*	1

### Biomarker evaluation

For POC tests, selected frozen samples were thawed at room temperature, and 100 μL of each sample was inoculated onto the AFIAS individual sample pouch for DD, IL‐6 and PCT plus G kits (AFIAS1, Boditech Med Inc.). Samples with concentrations above the upper detection limit of the assays or exhibiting high viscosity were appropriately diluted with saline solution (DD, 1:1000 and IL6, 1:100). The results were multiplied by the dilution factor. The results were obtained after 12 min.

For conventional laboratory analysis, 200 μL of SF was used, and all measurements were conducted using a Cobas 8000 analyser (Roche Diagnostics). Similar to the POC tests, the samples were diluted as needed. Through conventional analysis, DD results were obtainable within 12 min, whereas IL6 and PCT required 21 min.

### Data analyses

A formal priori sample size calculation was not conducted. The study included all consecutive eligible revision arthroplasties with available SF samples recorded in the institutional registry and biobank during the study period. Frequencies were computed for categorical variables, whereas medians with interquartile ranges were calculated for continuous variables, as appropriate. Pearson's chi‐squared test and Mann–Whitney *U*‐test were employed to compare clinical characteristics and biomarker findings between infected and noninfected cases for categorical and continuous variables, respectively. The diagnostic accuracy of all biomarkers was evaluated using the area under the receiver operating characteristic curve, sensitivity, specificity, positive predictive value and negative predictive value. Only the infection confirmed (*n* = 123) and infection unlikely (*n* = 145) groups were used for the diagnostic accuracy calculations. The optimal cutoff values were determined using *Youden's* index. Pairwise comparisons of the area under the curves (AUCs) were adjusted for multiple tests using Bonferroni correction. Statistical significance was set at *p* < 0.05. All analyses were conducted using the Statistical Package for Social Sciences 25 (IBM Corp., Released 2018, IBM SPSS Statistics for Windows, Version 25.0).

## RESULTS

### Diagnostic accuracy of biomarkers

Among the POC tests, synovial IL‐6 showed the highest AUC 0.83 (95% CI 0.77–0.89) while PCT performed poorly (0.26, 95% CI 0.20–0.33) (Tables 3 and 4). In laboratory methods, synovial IL‐6 demonstrated the highest AUC (0.94, 95% CI 0.90–0.97). POC and laboratory methods showed significant differences for IL‐6 (*p* = 0.001) and DD (*p* < 0.001), but not for PCT (*p* = 0.725) (Table 5). Combined POC tests showed better performance than individual tests. IL‐6 and DD together showed the highest AUC (0.92, 95% CI 0.88–0.95). In laboratory test combinations, IL‐6 and DD (0.89, 95% CI 0.85–0.93) did not exceed IL‐6 alone (0.94, 95% CI 0.90–0.97) (Table 5).

**Table 3:** Diagnostic accuracy of synovial markers using both point‐of‐care testing and routine laboratory measurements. European Bone and Joint Infection Society diagnostic criteria used as the reference standard.

Tested synovial biomarker	AUC (95% CI)	Threshold	Sensitivity	Specificity	ppv	npv
IL‐6 (pg/mL): POC	0.83 (0.77–0.89)	7260.45	0.73	0.94	0.91	0.80
LAB	0.94 (0.90–0.97)	4355	0.81	0.94	0.93	0.85
PCT (ng/mL): POC	0.26 (0.20–0.33)	47.5	0.08	0.99	0.33	0.54
LAB	0.28 (0.22–0.35)	0.12	0.08	0.97	0.20	0.54
DD (ng/mL): POC	0.66 (0.59–0.72)	463,790	0.66	0.60	0.58	0.67
LAB	0.80 (0.74–0.85)	266,000	0.77	0.74	0.72	0.79
Combination of POC tests						
IL‐6 + DD	0.92 (0.88–0.95)		0.73	0.94	0.91	0.80
Combination of LAB tests						
IL‐6 + DD	0.89 (0.85–0.93)		0.69	0.92	0.88	0.78

Abbreviations: AUC, area under the curve; CI, confidence interval; DD, D‐dimer; IL‐6, interleukin 6; LAB, laboratory measurement; npv, negative predictive value; PCT, procalcitonin; POC, point‐of‐care; ppv, positive predictive value.

**Table 4:** True and false negative and positives by using both point‐of‐care testing and routine laboratory measurements. European Bone and Joint Infection Society diagnostic criteria used as the reference standard.

	IL‐6 POC	IL‐6 LAB	PCT POC	PCT LAB	DD POC	DD LAB
True negative	135	135	141	140	86	106
False negative	33	23	122	121	42	28
False positive	9	8	2	4	58	37
True positive	90	99	1	1	81	94
Total^a^	267	265	266	266	267	265

Abbreviations: DD, D‐dimer; IL‐6, interleukin 6; LAB, laboratory measurement; PCT, procalcitonin; POC, point‐of‐care.

a Of the 268 samples tested, a few failed to yield results (IL‐6 POC: *n* = 1, IL‐6 LAB: *n* = 3, PCT POC: *n* = 2, PCT LAB: *n* = 2, DD POC: *n*: *n* = 1, DD Lab: *n* = 3).

**Table 5:** Pairwise comparison of the area under the curve for synovial fluid point‐of‐care tests and lab tests (alone and in combination).

	Difference between AUCs	95% CI	*p*‐Value
**POC and LAB tests**			
IL_6_POC versus IL_6_LAB	−0.101	−0.161 to 0.041	0.001
PCT_POC versus PCT_LAB	0.014	−0.064 to 0.092	0.725
DD_POC versus DD_LAB	−0.138	−0.205 to 0.072	0.000
**POC tests**			
IL_6 and DD versus IL_6	0.089	0.042–0.136	0.000
IL_6 and DD versus DD	−0.256	−0.318 to 0.194	0.000
IL_6 versus DD	−0.167	−0.262 to 0.073	0.001
**LAB tests**			
IL_6 and DD versus IL_6	−0.041	−0.066 to 0.016	0.001
IL_6 and DD versus DD	−0.098	−0.134 to 0.063	0.000
IL_6 versus DD	−0.139	−0.189 to 0.089	0.000

Abbreviations: AUC, area under the curve; CI, confidence interval; DD, D‐dimer; IL‐6, interleukin 6; LAB, laboratory measurement; PCT, procalcitonin; POC, point‐of‐care.

### Distribution of biomarkers in various clinical scenarios

Among septic revisions, no markers showed significant differences between culture‐negative and culture‐positive procedures (Table 6). Marker levels were significantly higher in high‐virulent than in low‐virulent organisms, except for IL‐6 POC (*p* = 0.631), DD POC (*p* = 0.080) and DD laboratory (*p* = 0.053) (Table 7). In 28 procedures with metallosis or polyethylene abrasion, DD showed the highest false‐positive rate (64%), while PMN% was the lowest (7%) (Table 8). Among the 123 PJI cases, 18 showed negative results for serum CRP, synovial WBC count and PMN%. IL‐6 laboratory and POC tests were positive in 16 and 14 cases, respectively (Table 9).

**Table 6:** Distribution of biomarkers in infected cases with unexpected negative intraoperative culture results.

Serum and synovial inflammatory markers (median and IQR)	UNIC (*n* = 23)	Culture positive (*n* = 100)	*p*‐Value
Serum CRP (mg/L)	36.6 (20.2–103)	73.9 (22.4–160.5)	0.352
Synovial WBC count (cells/µL)	19,180 (4280–51,690)	35,140 (8492.5–74,480)	0.240
Synovial PMN (%)	87.8 (78.2–91)	88.7 (81.4–95.1)	0.215
Synovial IL6_POC	14,866 (1965–25,234)	25,234 (5512–25,234)	0.802
Synovial IL6_LAB	18,300 (7870–55,500)	19,500 (5860–77,000)	0.670
Synovial PCT_POC	0.01 (0.01–0.03)	0.01 (0.01–0.02)	0.347
Synovial PCT_LAB	0.01 (0.01–0.02)	0.01 (0.01–0.02)	0.699
Synovial DD_POC	457,600 (351,270–951,040)	706,540 (404,173–2,181,583)	0.147
Synovial DD_LAB	573,000 (362,000–788,000)	481,000 (235,000–78,1000)	0.295

Abbreviations: DD, D‐dimer; EBJIS, European Bone and Joint Infection Society; ICM 2018, International Consensus Meeting 2018; IL6, interleukin 6; LAB, laboratory measurement; PCT, procalcitonin; PMN, synovial polymorphonuclear leucocytes; POC, point‐of‐care; WBC, synovial white blood cell.

**Table 7:** Distribution of biomarkers in infected cases according to pathogen virulence.

Serum and synovial inflammatory markers (median and IQR)	Low virulent (*n* = 40)	High virulent (*n* = 60)	*p*‐Value
Serum CRP (mg/L)	20 (7.8–50.9)	115.2 (69.3–219.8)	0.000
Synovial WBC count (cells/µL)	13,900 (2920–26,900)	58,950 (30,700–84,460)	0.000
Synovial PMN (%)	89.3 (79.1–95.6)	88.5 (83–94.8)	0.759
Synovial IL6_POC	25,232 (4031–25,232)	25,234 (8381–25,234)	0.631
Synovial IL6_LAB	11,050 (5395–28,150)	45,800 (9430–136,100)	0.009
Synovial PCT_POC	0.01 (0.01–0.01)	0.01 (0.01–0.03)	0.035
Synovial PCT_LAB	0.01 (0.01–0.02)	0.01 (0.01–0.01)	0.242
Synovial DD_POC	536,505 (400,097–1,100,143)	908,885 (445,322–2,667,193)	0.080
Synovial DD_LAB	359,500 (400,098–574,750)	592,000 (182,000–869,000)	0.053

Abbreviatioins: DD, D‐dimer; EBJIS, European Bone and Joint Infection Society; ICM 2018, International Consensus Meeting 2018; IL6, interleukin 6; LAB, laboratory measurement; PCT, procalcitonin; PMN, synovial polymorphonuclear leucocytes; POC, point‐of‐care; WBC, synovial white blood cell.

**Table 8:** Distribution of biomarkers in metallosis or polyethylene abrasion cases.

Biomarker	Median and IQR	Number of procedures with biomarker levels above the infection cutoff		
		Total (*n* = 28)	Not infected (*n* = 14)	Inconclusive (*n* = 14)
Serum CRP	4 (1.5–17.2)	11 (39)	3	8
Synovial WBC count	1630 (453–4138)	8 (29)	0	8
Synovial PMN (%)	23.5 (13.5–50.9)	2 (7)	0	2
Synovial IL6_POC	5757.6 (896.3–25,234)	12 (43)	4	8
Synovial IL6_LAB	1410 (697.5–7892.5)	13 (46)	4	9
Synovial PCT_POC	0.275 (0.01–0.05)	1 (13)	1	0
Synovial PCT_LAB	0.01 (0.01–0.04)	1 (13)	0	1
Synovial DD_POC	474,170 (306,010–583,840)	14 (50)	4	10
Synovial DD_LAB	364,000 (173,750–615,000)	18 (64)	6	12

Abbreviations: DD, D‐dimer; IL6, interleukin 6; LAB, laboratory measurement; PCT, procalcitonin; PMN, synovial polymorphonuclear leucocytes; POC, point‐of‐care; WBC, synovial white blood cell.

**Table 9:** Performance of the synovial point‐of‐care tests in infected cases with serum C‐reactive protein, synovial white blood cell count and polymorphonuclear leucocyte percentage below the infection threshold.

	Total (*n* = 18)
**Joint type, *n* (%)**	
Hip	15 (83)
Knee	3 (17)
**Raised serum and synovial inflammatory markers, *n* (%)**	
Serum CRP (mg/L)	0
Synovial WBC count (cells/µL)	0
Synovial PMN (%)	0
Synovial IL6_POC	14 (78)
Synovial IL6_LAB	16 (89)
Synovial PCT_POC	0
Synovial PCT_LAB	0
Synovial DD_POC	10 (56)
Synovial DD_LAB	14 (78)
**On antibiotic therapy at the time of aspiration, *n* (%)**	7 (39)
Serum CRP (mg/L)	0
Synovial WBC count (cells/µL)	0
Synovial PMN (%)	0
Synovial IL6_POC	6
Synovial IL6_LAB	7
Synovial PCT_POC	0
Synovial PCT_LAB	0
Synovial DD_POC	3
Synovial DD_LAB	7

Abbreviations: DD, D‐dimer; IL6, interleukin 6; LAB, laboratory measurement; PCT, procalcitonin; PMN, synovial polymorphonuclear leucocytes; POC, point‐of‐care; WBC, synovial white blood cell.

## DISCUSSION

The most important finding of this study was that SF POC IL‑6 levels demonstrated good diagnostic accuracy for PJI. Furthermore, the combination of POC IL‐6 with DD enhanced diagnostic accuracy, although both methods remained less effective than standard laboratory techniques.

Among the evaluated POC tests, synovial IL‐6 showed the highest diagnostic accuracy, consistent with previous studies that have demonstrated the utility of IL‐6 in PJI diagnosis, although primarily through laboratory‐based assays [16, 17, 31, 32]. Despite its lower accuracy than that of laboratory methods, POC IL‐6 testing could be valuable for rapid decision‐making in outpatient clinics for initial screening or in intraoperative settings to guide the surgical management of patients with PJI. In contrast, the diagnostic utility of other tested POCs, particularly PCT, was lower, consistent with prior reports that questioned the value of PCT in the context of PJI [4, 26].

Notably, laboratory‐based IL‐6 outperformed all other markers, further supporting its role as a robust diagnostic biomarker for PJI [20, 31, 32]. In an initial IL‐6 POC study, which included only a small sample size (*n* = 26), Wimmer et al. reported a strong correlation between IL‐6 POC and laboratory measurements [29]. However, the significant difference in AUCs between POC and laboratory IL‐6 and DD tests in the present study suggests that POC methods require further calibration and validation for use with complex SF matrices. Additionally, we observed a lower AUC for DD, which contrasts with the sole available study on synovial DD, which reported an AUC of 0.99 [13]. This discrepancy may be due to the fact that the majority (52/69) of patients in that study were control patients with primary osteoarthritis who underwent TKA.

Several studies have demonstrated that combining biomarkers enhances diagnostic accuracy, a finding that we also observed with the IL‐6 and DD combination [3, 7, 8, 10, 27]. In contrast, Klim et al. reported no benefit from combining serum biomarkers, whereas by combining serum and synovial markers, Yu et al. found better performance than serum markers alone, but in comparison to synovial markers, either similar or lower performance was obtained [14, 32]. Similarly, in the laboratory settings of the present study, the addition of DD and PCT to IL‐6 did not improve the diagnostic performance of IL‐6 alone. This observation highlights the lower accuracy of POC tests, likely due to assay interference from the sample matrix, which these tests cannot mitigate as efficiently as laboratory tests. This weakness of POC tests may be compensated for by using a second independent biomarker. The present results support laboratory‐based synovial IL‐6 testing wherever it is logistically feasible, emphasising the importance of context‐specific diagnostic approaches.

Studies assessing the efficacy of biomarkers have mostly focused on septic and aseptic PJIs [4, 9, 32]. In this study, we evaluated the applicability of these biomarkers in complex clinical scenarios to determine their practical utility. Our findings revealed no significant differences in biomarker levels between culture‐positive and culture‐negative infections, underscoring the potential of these markers for diagnosing PJIs, even in the absence of microbiological confirmation of infection. Consistent with the existing literature, all biomarkers were significantly elevated in infections caused by high‐virulent organisms, with the exception of POC and laboratory DD and POC IL‐6, suggesting the relevance of these markers in infections caused by low‐virulent pathogens [6].

In the present study, DD exhibited a high false‐positive rate in patients with metallosis or polyethylene wear, limiting its specificity in these contexts. This aligns with previous reports indicating that metal debris and associated inflammatory responses in metallosis can elevate biomarker levels independently of infection [2, 7]. However, the absence of prior studies utilising synovial DD makes it difficult to draw comparisons or determine its efficacy. Interestingly, in these complications, synovial PMN% was found to be the most reliable biomarker, which is in line with a previous study by Abdelaziz et al. [1]. These findings underscore the importance of considering the strengths and limitations before selecting the most appropriate diagnostic marker.

In confirmed PJIs with normal serum CRP and synovial cell counts, IL‐6 identified most cases, underscoring its utility as a sensitive marker in diagnostically ambiguous situations, including those with prior antibiotic exposure. The cost of conducting IL‐6 or DD POC in the present study was approximately €14, which may vary depending on the type or make of the kit, yet remains less expensive than other tests, such as alpha defensin (€300–€500) or calprotectin (€150–€250). Additionally, low‐cost POC biomarkers, such as synovial IL‐6 combined with DD, provide rapid diagnostics that reduce PJI evaluation costs while maintaining their diagnostic accuracy. These cost considerations are particularly relevant in resource‐constrained settings and high‐volume arthroplasty centres.

From a clinical standpoint, SF POC testing may be useful in outpatient clinics and intraoperative settings, where rapid decision‐making is required, and immediate laboratory access is limited. In these settings, POC IL‐6 testing, either alone or in combination with DD, can supplement standard diagnostics and help detect infections that routine serum CRP and synovial cell counts may miss.

This study had several limitations. First, it was a single‐centre retrospective analysis of a referral population, which limits generalisability. Second, freezing and thawing can alter cellular and protein components, although previous studies on biomarkers, including DD, IL‐6 and PCT have employed frozen samples and shown that overall diagnostic accuracy remains reliable [4, 5, 7, 11, 15, 21, 25]. Nonetheless, further validation of our findings is required in large‐scale prospective studies using fresh SF samples to confirm their clinical applicability. Third, the POC assays used were off‐label adaptations of platforms designed for blood or serum, which may explain their lower performance than laboratory‐based assays. Furthermore, the necessity to dilute samples requires an adjustment in the linear range of the assays and an enhancement of the POC methodology for the markers being evaluated. Finally, this study did not directly compare these POC biomarkers with synovial leucocyte counts and differentials in a cost‐effectiveness framework. Future studies should include this to better define their role in routine PJI workup.

## CONCLUSIONS

This study supports the potential utility of SF POC tests, particularly IL‐6, for rapid diagnosis of PJI. The combination of IL‐6 with DD appears promising for enhanced POC diagnostic accuracy. The performance of IL‐6 and DD in challenging clinical scenarios underscores the need for tailored biomarker strategies. While the present findings are promising, further studies are essential to validate these results, standardise POC methods, and assess the real‐world impact of POC testing on clinical outcomes and resource utilisation before clinical implementation.

## AUTHOR CONTRIBUTIONS

**Sujeesh Sebastian**: Methodology; formal analysis; investigation; data curation; Writing—original draft preparation. **Hibah Abdulwahab Abusulaiman**: Methodology; investigation, data curation; writing—reviewing and editing. **Veronika Achatz**: Data curation; writing—reviewing and editing. **Matteo Spadini**: Data curation; writing— reviewing and editing; **Tobias Josef Niedrist**: Methodology; investigation; writing—reviewing and editing. **Markus Herrmann**: Supervision; writing—reviewing and editing. **Jochen Gerhard Hofstaetter**: Conceptualisation; supervision; writing—reviewing and editing.

## CONFLICT OF INTEREST STATEMENT

The authors declare no conflicts of interest.

## ETHICS STATEMENT

This study was approved by the ethics committee of the Vienna Hospitals of the Vinzenz Group (EC 04‐2021), and informed consent was obtained from all patients.

## Data Availability

The data that support the findings of this study are available on request from the corresponding author.
